# Fear of Loneliness: Development and Validation of a Brief Scale

**DOI:** 10.3389/fpsyg.2020.583396

**Published:** 2020-10-23

**Authors:** José Ventura-León, Andy Rick Sánchez-Villena, Tomás Caycho-Rodríguez, Miguel Barboza-Palomino, Andrés Rubio

**Affiliations:** ^1^Facultad de Salud, Escuela de Psicología, Universidad Privada del Norte, Lima, Peru; ^2^Facultad de Enfermería, Universidad Andres Bello, Santiago, Chile; ^3^Facultad de Psicología, Universidad Diego Portales, Santiago, Chile

**Keywords:** loneliness, solitude, belonging, instrument validation, isolation, fear

## Abstract

This research aims to develop and validate a Spanish version of The Brief Scale of Fear of Loneliness (BSFL). Participants were 1385 youth and adults, 347 from a pilot sample and 1032 from the final version, whose ages were in the range of 18 to 40 years. Two instruments, the Rosenberg Self-Esteem Scale and the De Jong Gierveld Loneliness Scale, in their Peruvian versions, were used to support the relationship with other variables. Results show that the BSFL should be interpreted as a one-dimensional measure, the same ones that were examined at the exploratory level and verified at the confirmatory moment (RMSEA < 0.08, CFI > 0.95), and its reliability is considered good (ω > 0.88). In addition, the quality of the item content was reviewed by six expert judges for relevance and validity, with Aiken’s V being greater than 0.70. It is concluded that the BSFL is a valid and precise short instrument that can be used in future research studies.

## Introduction

### Loneliness

It has been known for a long time that 71% of people between 18 and 24 years old report feeling lonely sometimes or often, and between 25 and 34 years old the figure drops slightly to 69% (Parlee, 1979). Recent figures reveal 17% of people between 18 to 24 years old and 25 to 35 years old said they felt alone quite often and very often, respectively, also experiencing anxiety and concern about feeling alone (YOUGOV, 2016). Likewise, according to a prevalence study carried out in the United Kingdom, it is known that women under 25 years of age present greater amounts of loneliness than men, with 9 and 6% respectively, claiming to always feel alone (Victor and Yang, 2012). Similar results are reported in university students in Amsterdam, where the prevalence of loneliness reaches 23% (Pijpers, 2017).

Loneliness is characterized by a lack of satisfaction in interpersonal relationships (Andersson, 1993), which arises when support and social networks are impaired (Perlman and Peplau, 1981), and the individual experiences feelings of isolation, not belonging, incomprehension, rejection (Rook, 1984), or lack of company (Francis, 1976). Furthermore, loneliness includes deficiencies, disagreements, isolation, and psychological pain manifested in sadness, boredom, and a feeling of emptiness (Stein and Tuval-Mashiach, 2015).

Loneliness has been linked to many other variables. For example, in the biological field, it has been observed that loneliness has been related to different variables such as blood pressure, cortisol levels, hypersensitivity to stressors and immunosuppression (Brown et al., 2017, 2019). From a clinical point of view, there is evidence of a relationship with suicidal ideation and behavior (Chang et al., 2017; Calati et al., 2018), depressive symptoms, and social anxiety (Lasgaard et al., 2011; Danneel et al., 2019). Regarding family related factors, a relationship has been found with the interaction with parents in their positive communication and time together (Majorano et al., 2017), with family cohesion and adaptability (Fujimori et al., 2017), and with stress in the academic context (Stoliker and Lafreniere, 2015). Finally, in the intra and interpersonal dimensions, it has been associated with shyness (Bian and Leung, 2015; Muyan et al., 2018), avoidance (Demirli and Demir, 2014), anxiety to speak (Odaci and Kalkan, 2010), and self-esteem (Chiao et al., 2019). On the other hand, loneliness has a mediating effect on the relationship between excessive use of social networks and real-life social interaction in university students (Ndasauka et al., 2016), as well as on the relationship between social skills and depressive and anxious symptoms (Moeller and Seehuus, 2019). Furthermore, it is known that loneliness is greater in men than in women (Wiseman et al., 1995), however, with small effect sizes (Maes et al., 2019).

Despite the fact that loneliness has been studied in relation to many variables from various fields, there are no studies that explore fear of the experience of loneliness *per se*, which has been considered for a time as painful and acute in young and adult population, more than in other ages (Rokach, 2000).

### Fear of Loneliness

Fear is an unpleasant experience that implies a degree of awareness of the individual (Uribe et al., 2007), with its etymology being associated with the suspicion of future danger or harm (Real Academia and Española, 2014). Despite this, the term fear can be understood as an attitude toward something (Uribe et al., 2007; Simkin and Quintero, 2017) that triggers behaviors of fight, escape, or avoidance (Bay and Algase, 1999) to the topic, situation or place (Wong et al., 1997), that are products of the beliefs that the individual experiences and generates, and have an impact in their daily life (Al-Namankany et al., 2012). Nonetheless, the terms fear and anxiety are used interchangeably (Tomás-Sábado, 2016), although the latter has a more cognitive component (Whitley, 1992; Catherall, 2003), while the first one is more behavioral in nature (Blanchard and Blanchard, 1990a,b). Despite this, there is a reciprocal interaction between them (Bandura, 1977).

In this sense, the Fear of Loneliness (FL) can be understood as an attitude of avoidance accompanied by worrying thoughts and feelings of abandonment that the individual experiences when she/he is alone. This definition can be used to interpret the scores on the scale.

The notion of studying the responses of fear toward a specific object is not new since it is known that there are scales that measure fear of negative evaluation (Gallego et al., 2007; Zubeidat et al., 2007), of death (Tomás-Sábado et al., 2007; López and Calle, 2008), flying (Dongil and Wood, 2009), and even fear of dental treatment (Armfield, 2010; Al-Namankany et al., 2012; Ibrahim et al., 2017). Therefore, it is not strange to conceptualize loneliness as a specific fear.

The problem of loneliness takes on particular relevance in the current context, considering aspects such as the lack of sense of belonging linked to the increasing levels of individualism (Santos et al., 2017). In addition, the phenomenon of sustained growth in the use of social networks must also be considered, which in some cases is associated with the feeling of loneliness (Song et al., 2014). In this sense, and considering that the feeling of loneliness has been shown to be associated with self-esteem in many different populations (Creemers et al., 2012; Domagała-Krecioch and Majerek, 2013; Kong and You, 2013; Tian, 2016), it could be thought that the fear of loneliness today plays a key role in people’s well-being, as well as in the constitution of their identities. Due to the above, it is necessary to design and validate instruments that measure this particular construct.

### The Present Research

There are various instruments that have measured loneliness since the 1970s (Russell et al., 1978), 80s (Russell et al., 1980; De Jong-Gierveld and Kamphuls, 1985), 90s (Russell, 1996; Cramer and Barry, 1999) as well as periods after 2000 (Hughes et al., 2004; Maes et al., 2015). Despite this, there is no instrument that measures FL; with the closest being the Fear Survey Schedule (Rubin et al., 1969). Although these authors developed a scale with 122 items, five of which are related to fear of isolation or loneliness, they do so in a general way, without considering criteria that allow addressing the phenomenon in a more specific and complete way. In this context, the purpose of the study is to develop and validate a Brief Scale of FL in youth and adults, considering the content-based evidence through expert judgment, testing the internal structure of the scale through exploratory and confirmatory analysis, the calculation of the reliability using the omega coefficient and the establishment of the invariance according to sex.

## Materials and Methods

### Participants

Participants were 1385 youth and adults split in two groups. The first one was made up of 347 people between 18 and 40 years old (Average = 23.26; *SD* = 6.51), with 204 being women and 143 men. With these participants the EFA was performed. The participants were university students from the faculties of Health Sciences (49.86%), Business (20.75%), Engineering (12.97%), Architecture and Design (6.63%) and Rights and Political Sciences (4.03%), which were in academic cycles from 1st to 10th. The second group consisted of 1,032, whose ages also ranged from 18 to 40 years (Mean = 21.09; *SD* = 3.38), with 795 being women and 237 men, and whose responses were used for the CFA. It is worth mentioning that these young people and adults were university students. From the faculties of Health Sciences (89.44%), Engineering (3.88%), Administration (2.62%), Architecture and Design (1.55%), Business (1.16%), Law and political science (1.07%), and Communications (0.3%). All the participants were people residing in the northern area of Metropolitan Lima.

### Instruments

#### The Brief Scale of Fear of Loneliness (BSFL)

It is made up of five items belonging to a single factor. Possible responses indicated frequency, where 0 = Never; 1 = Rarely; 2 = Sometimes; 3 = Almost always; and 4 = Always. Psychometric properties are the object of the present study.

#### The Rosenberg Self-Esteem Scale ([RSE]; Rosenberg, 1965)

It made up of 10 items, distributed into five negative or inverse items, and five positive items, with a Likert scale ranging from 1 to 4. The psychometric properties of the RSE in Peru were adequate (Ventura-León et al., 2018).

#### The De Jong Gierveld Loneliness Scale ([DJGLS]; De Jong-Gierveld and Kamphuls, 1985)

It is a scale made up of 11 items with a scale ranging from 0 to 3. However, the answers must be dichotomized, so that the DJGLS reaches a maximum score of 11 points. Psychometric properties in Peru were analyzed by Ventura-León and Caycho (2017).

### BSFL Construction Procedures

For the construction of the BSFL, the recommendations of the International Test Commission were followed (International Test Commission [ITC], 2018). Initially, different databases (Redalyc, Scielo, Scopus, Proquest, Google Scholar, Taylor and Francis, Sage) were reviewed in order to search for different theories where FL is addressed. First, the variable was operationalized by means of a specification table where the definition was set and five items emerged. Second, the five items were submitted to the scrutiny of six expert judges who rated the relevance and validity of the items on a scale of 0 (not at all) to 3 (totally); furthermore, the responses were quantified using Aiken’s V (Ventura-León, 2019). Third, the BSFL was applied to a pilot sample of 347 people who signed an informed consent and then answered the scale; their answers were subjected to an exploratory factor analysis to verify the quality of the items and if they belong in the factor (Ferrando and Lorenzo-Seva, 2014). Finally, the scale was applied to 1,032 participants, whose responses were subjected to confirmatory factor analysis and other statistical techniques for information processing. In this final application, two tests validated in Peru are incorporated. One, about Loneliness to examine the incremental validity, because it is known that in the face of new measures it is necessary to evaluate its functioning with an available alternative that measures the same or something similar (Hunsley and Meyer, 2003) and a self-esteem scale also validated in Peruvian context to examine the relationship with another variable and provide evidence of the predictive capacity of the BSFL (American Educational Research Association [AERA] et al., 2014).

### Analysis of Data

Statistical analyses were performed with two open access programs: FACTOR version 10.9 for exploratory factor analysis (EFA) and Rstudio version using the ‘lavaan’ library (Rosseel et al., 2018). In the first stage, a preliminary analysis of the items was carried out considering kurtosis and asymmetry, where values greater than ± 1.5 would reflect a distortion of normality (Ferrando and Anguiano-Carrasco, 2010).

In the second stage, the dimensionality analysis of the scale was carried out in two modalities: (a) EFA, for which it was necessary to review the sample adequacy measures (KMO and Bartlett’s sphericity test). The estimation method was robust unweighted least squares (RULS) with a matrix of polychoric correlations by the ordinality of the data, and for the determination of the number of factors, parallel analysis was used, a simulation technique that compares random values with empirical values (Timmerman and Lorenzo-Seva, 2011); (b) confirmatory factor analysis (CFA) where the following adjustment indices were used (Hu and Bentler, 1999): χ^2^, Root Mean Square Error of Approximation (RMSEA < 0.06), Weighted Root Mean Square Root (WRMR < 1), Comparative Adjustment Index (CFI > 0.95) and Tucker-Lewis Index (TLI > 0.95). In addition, the estimator was Weighted Least Square Mean and Variance Adjusted (WLSMV) because it was ordinal data (Brown, 2015). In both types of factor analysis (EFA and CFA), the belonging of an item to a factor was determined by factor loadings greater than or equal to 0.30 (Kline, 2015).

In a third stage, reliability was estimated using the omega coefficient (ω) that reflects the proportion of common variance shared by the items (Ventura-León and Caycho-Rodríguez, 2017), where values above 0.70 are considered recommendable.

In the fourth stage, factor invariance according to sex was calculated under the recommendations of Wu and Estabrook (2016), who point out that in the case of ordinal data the invariance cannot be examined only by restricting a set of parameters at a time. In this way, configural invariance (base model), metric invariance (thresholds, loading constrained to be equal across groups), scalar invariance (thresholds, loadings, intercepts) and strict invariance (thresholds, loadings, intercepts, residual) were tested. Theta –parameterization was used which allows the calculation of the residuals (strict invariance). Also, the WLSMV was used as an estimator (Brown, 2015). To observe the suitability of the invariance, minimal differences were established between the two models according to those presented by Finch and French (2018), who point out that an RMSEA (ΔRMSEA) < 0.01 is adequate for ordinal variables and what Chen (2007) postulated, where CFI (ΔCFI) ≤ 0.01 is adequate. It is worth mentioning that this continuous cut-off point is used because it does not have a categorical version, as there are minimal differences between the models.

The fifth stage consisted of contrasting the evidence in relation to other variables. Therefore, correlations were modeled from SEM with the De Jong Gierveld loneliness scale (Ventura-León and Caycho, 2017) and Rosenberg’s Self-esteem (Ventura-León et al., 2018), following Cohen’s recommendations for the magnitude of the effect (2009) where: *r* ≥ 0.10 is small; *r* ≥ 0.30 is considered moderate; *r* ≥ 0.50 shows a strong effect.

## Results

### Evidence Based on Content

The analysis of the content of the items was carried out by expert judges who rated the scale based on two criteria: (a) Representativeness, indicating the correspondence between the content of the items and the definition of the construct, and (b) Relevance, the importance of including the items on the scale. An Aiken’s V of 1.00 is observed for all items in the two criteria, suggesting evidence about the content of the BSFL.

### Preliminary Analysis of the Items

As seen in Table 1, items 4 and 5 have a higher arithmetic mean. All items show kurtosis and asymmetry values below ± 1.5 (Ferrando and Anguiano-Carrasco, 2010) with positive kurtosis, except for item 4. Additionally, the Mardia coefficient was calculated, which presented a value of 45.98, which indicates little deviation from multivariate normality.

**TABLE 1:** Descriptive Statistics of the BSFL.

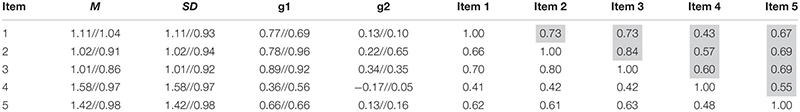

*From the//symbol to the left EFA and to the right CFA; From the diagonal down the matrix of the EFA and upward of the CFA. M, mean; *SD*, standard deviation; g1, asymmetry; g2, kurtosis.*

### Evidence Based on Internal Structure

For the EFA (Table 2), RULS were used, with a polychoric correlation matrix and Parallel Analysis (PA) with optimal implementation (Timmerman and Lorenzo-Seva, 2011). The KMO test (0.83) and Bartlett’s sphericity test [χ^2^(10) = 743.9, *p* < 0.001] indicated that it is possible to carry out an EFA (Abad et al., 2012).

**TABLE 2:** Standardized factor loadings of the BSFL in the exploratory factor analyses.

Item	F1	*h*^2^
(1) I fear someone may leave me [*Temo que alguien pueda abandonarme*]	0.80	0.64
(2) The idea of being alone worries me [*La idea de estar solo me preocupa*]	0.85	0.73
(3) I am afraid of being alone [*Tengo miedo a estar solo*]	0.88	0.77
(4) When I am alone, I look for someone’s company [*Cuando estoy solo, busco la compañía de alguien*]	0.52	0.27
(5) I am concerned that someone is leaving my side [*Me preocupa que alguien se aleje de mi lado*]	0.76	0.57
ω	0.88	
Load/h^2^ mean	0.76/0.59	
Eigenvalue	3.33	
% of Variance	66.68	

**h*^2^ = communality; F1 = Fear of loneliness.*

The PA pointed out the existence of a single underlying factor, which explains 66.68% of the total variance, with an Eigenvalue of 3.33. Goodness of fit indices were excellent (χ^2^ = 10.70, df = 5, *p* = 0.060; RMSEA = 0.06, CFI = 1.00. WRMR = 0.05). It should be noted that robust Chi square was used with mean and scaled variance (Asparouhov and Muthen, 2010).

**FIGURE 1:**
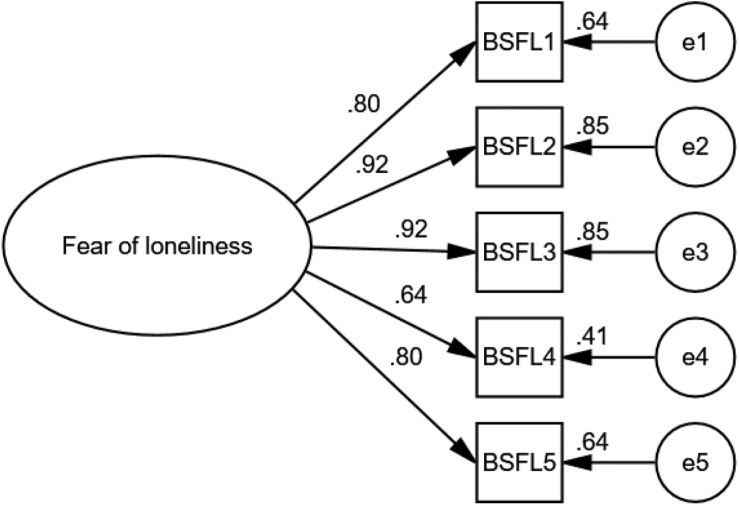
Factorial structure of the BSFL.

The CFA (Figure 1) was carried out with a second group of participants (*N* = 1032) in order to verify what was obtained in the EFA. A single factor structure was modeled that revealed good goodness of fit (χ^2^ = 12.93, df = 5, χ2/df = 2.59, RMSEA = 0.04, CFI = 1.00, TLI = 1.00).

### Reliability

Reliability was calculated for each of the moments of the factor analysis. Thus, at the exploratory level the omega coefficient is considered good (ω = 0.88) and at the confirmatory level considered excellent (ω = 0.91). In this way, it can be seen that the factor loads are strong.

### Factor Invariance According to Sex

Table 3 shows the measurement invariance, which was evaluated starting from a base model called configural invariance (M1), metric (M2), scalar (M3), strict (M4), the estimator used WLSMV was robust because it considers ordinal variables (Brown, 2015). In this sense, the base model examined the fit in both groups without restrictions on some of the parameters. Then, the M2 model, which is a model where the loads and thresholds of each item so that they have the same value in men and women. It is seen that the difference between M1 and M2 are minimal (ΔCFI < 0.01), accepting the hypothesis that the thresholds are invariant. Subsequently, invariance in loads, thresholds and intercepts is examined adding restrictions on all items across subgroups (M3). It is observed that the difference between M3 and M1 is minimal (ΔCFI < 0.01). Finally, the strict invariance is evaluated where the loads, thresholds, intercepts and residuals are the same in the two groups, examining that the differences are within expectations (ΔCFI < 0.01).

**TABLE 3:** Analysis of factor invariance according to the gender of the BSFL.

Models	χ^2^(*df*)	Δχ^2^	Δ*df*	*p*	CFI	ΔCFI
M1	31.88 (10)	-	-	-	-	-
M2	37.17 (20)	7.68	10	0.011	0.99	0.001
M3	34.58 (24)	1.25	4	0.075	0.99	0.002
M4	41.13 (28)	5.88	4	0.052	0.99	−0.001
M5	49.09 (33)	6.78	5	0.035	0.99	−0.001

*M1: configural; M2: threshold; M3: metric; M4: scalar; M5: strict.*

In view of the fact that factorial invariance was achieved, we proceeded to examine the differences according to sex through the latent means. In relation to the fear of loneliness, it is observed that women (*M* = 0.85) present a slightly higher value than men (*M* = 0.81); although it is not statistically significant and the effect size is trivial [*t*(405.89) = 0.70, *p* = 0.482, *d* = 0.05].

### Incremental Validity

**FIGURE 2:**
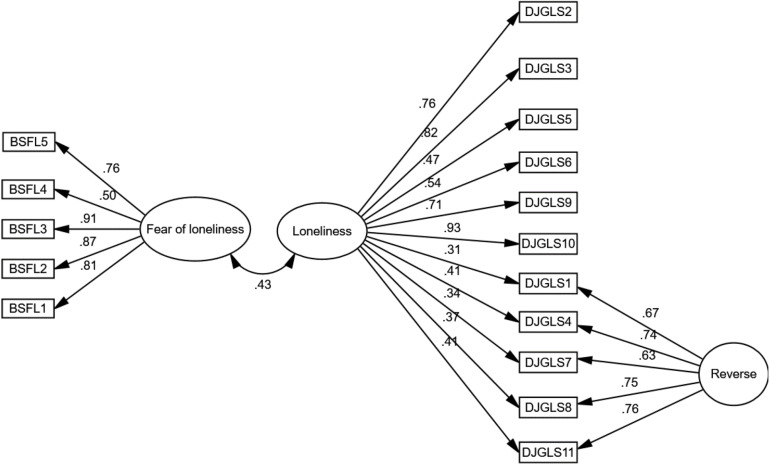
Structural model of the relationship between the BSFL and DJGLS.

The incremental validity of the BSFL was evaluated with a similar test that was validated in the Peruvian context, such as the DJGLS (De Jong-Gierveld and Kamphuls, 1985). In this sense, the results (Figure 2) show a moderate relationship (*r* = 0.43, CFI = 0.93; RMSEA = 0.06; SRMR = 0.08); which is good, because if the correlation is very high it would imply a conceptual overlap.

### Evidence Based on the Relationship With Other Variables

**FIGURE 3:**
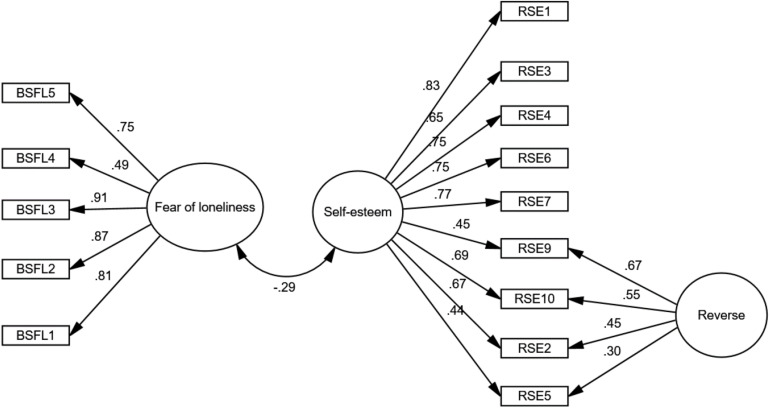
Structural model of the relationship between BSFL and RSE.

To evaluate convergence with other tests, a concurrency method was used. In this sense, the scores of the BSFL of the participants were correlated with the RSE (Rosenberg, 1965). The results showed (Figure 3) that RSE was negatively correlated with BSFL (*r* = −0.29, CFI = 0.94; RMSEA = 0.07; SRMR = 0.07). In this way, the predictive power of the BSFL is revealed.

## Discussion

Loneliness is a frequent event in youth and adulthood and for many years it has been known that the prevalence reaches 69% (Parlee, 1979) and that there is a concern about feeling alone in this age group (YOUGOV, 2016). Although there are various instruments that have measured loneliness since the 1970s (Russell et al., 1978, 1980; De Jong-Gierveld and Kamphuls, 1985; Russell, 1996; Cramer and Barry, 1999; Hughes et al., 2004; Maes et al., 2015). There is no instrument that assesses the fear of loneliness. In this sense, the main objective of the present study was to develop and validate a Brief Scale of Fear of Loneliness in Young People and Adults.

Regarding the evidence of validity, the content of the items was reviewed through the judgment of experts who found high representativeness and relevance of the items in the BSFL, which is often a forgotten action, but one that is necessary (International Test Commission [ITC], 2018). This accompanied by the Aiken’s V coefficient (Ventura-León, 2019) allowed verifying the quality of the items.

Regarding its internal structure, the BSFL is a one-dimensional measure of fear of loneliness. Thus, the EFA produced with polychoric correlation matrices by the ordinality of the data, revealed that a single factor underlies the five items, which explains 66.68% of the variance of the construct. Likewise, their factor loads were > 0.30 (Kline, 2015). These findings were corroborated by the CFA, which showed excellent goodness of fit indices (Hu and Bentler, 1999). Similarly, the assumption of one-dimensionality is consistent with theoretical arguments of previous studies regarding fear of a specific object (Zubeidat et al., 2007; Dongil and Wood, 2009; Ibrahim et al., 2017).

Regarding the reliability of the BSFL, it revealed good values both in its exploratory version (ω = 0.88) and confirmatory (ω = 0.91). These results are consistent with cut-off points established by various authors who suggest that values > 0.70 are considered acceptable (Ventura-León and Caycho-Rodríguez, 2017).

In relation to incremental validity, a similar test with Peruvian validation was used, as it is a recommendation by Hunsley and Meyer (2003). This in order to observe that if there is a relationship between both tests, that it is not large enough to suppose a conceptual overlap (American Educational Research Association [AERA] et al., 2014). These findings reveal that the BSFL measures something similar to the DJGLS, but not the same; therefore, they are constructs that are associated, but not the same.

To provide evidence based on the relationship with other variables, the convergence of the BSFL was evaluated, which demonstrated its predictability by presenting a moderate correlation (*r* = 0.29;Cohen, 1988) with the loneliness scale adapted to the Peruvian context (Ventura-León and Caycho, 2017) and the self-esteem scale (Ventura-León et al., 2018) showing an expected theoretical direction. Thus, the correlation between loneliness and self-esteem is consistent with previous studies; evidencing that experiencing fear of loneliness is associated in a moderate way with the self-assessment of the self (Brewer and Kerslake, 2015; Błachnio and Przepiorka, 2019; Chiao et al., 2019).

It is important to note that the present study reviewed the gender invariance of the BSFL. The measurement invariances (configurational, thresholds, and factor loads) were stable across the groups. In this sense, the items measure the latent trait in men and women in the same way (Brown, 2015), which is a requirement of the measurement instruments for comparison by groups (Byrne, 2008). Therefore, the one-dimensional structure of the BSFL according to sex, indicates that in the sample of Peruvian youth and adults (men and women) they conceptualize FL in an equivalent way, a situation that suggests that the factor structure is similar in both groups and that the differences between men and women are real and not the product of a measurement bias.

Regarding the theoretical implications, having this scale will allow evaluating theoretical models and seeing the relationship that the fear of loneliness (and not only the feeling of loneliness) has with well-being, the sense of belonging and with the construction of identity of individuals, in a society that becomes more and more individualistic (Santos et al., 2017) and consumer of social networks, which, in some cases, increase people’s feelings of loneliness (Song et al., 2014). Regarding the practical implications, the scale developed, due to its reduced number of items, allows a pragmatic and rapid measurement of the fear of loneliness in large populations, which would allow to orient preventive interventions on the psychosocial variables commonly associated with loneliness (drug use, excessive use of the internet, for example).

The study has some limitations. First, more participants are required in the invariance analysis for further studies. Second, it was not feasible to review the temporal stability of the scale; so, a test–retest is recommended in future studies. Third, the participants were people with higher studies, so the scale can only be interpreted for a population with similar characteristics; it is recommended to explore its operation in other populations.

It is concluded that the BSFL is a measure that has evidence of validity, reliability in its scores, being invariant regarding sex and predicting other behaviors quite well. Therefore, it is a short self-reported measure, easy and quick to apply, which will be useful in future research studies.

## Data Availability Statement

The raw data supporting the conclusions of this article will be made available by the authors, without undue reservation.

## Ethics Statement

Ethical review and approval was not required for the study on human participants in accordance with the local legislation and institutional requirements. The patients/participants provided their written informed consent to participate in this study.

## Author Contributions

JV-L involved in planning and supervised the work, processed data, performed the analysis, drafted the manuscript, and designed the figures. AS-V contributed in preparation of the published work, specifically critical review, commentary. TC-R and MB-P performed the measurements, sample design, aided in interpreting the results, and worked on the manuscript. AR contributed in presentation of the published work, specifically writing the initial draft (including substantive translation). All authors discussed the results and commented on the manuscript.

## Conflict of Interest

The authors declare that the research was conducted in the absence of any commercial or financial relationships that could be construed as a potential conflict of interest.
